# Durable response to osimertinib in an advanced lung adenocarcinoma patient with an uncommon *EGFR* T854A mutation: A case report

**DOI:** 10.1097/MD.0000000000031875

**Published:** 2022-12-09

**Authors:** Nan Zhao, Hua Xin, Changjuan Qin, Zhiqi Li, Hongbin Sun

**Affiliations:** Department of Thoracic Surgery, China-Japan Union Hospital of Jilin University, Changchun, China.

**Keywords:** case report, *EGFR* T790M, *EGFR* T854A, next generation sequencing, osimertinib

## Abstract

Epidermal growth factor receptor (*EGFR*) T854A mutation in exon 21 is an uncommon *EGFR* mutation in patients with non-small cell lung cancer (NSCLC). It is a secondary *EGFR* mutation after first- and second-generation EGFR tyrosine kinase inhibitors (TKIs). All *EGFR* T854A mutations were co-occurred with *EGFR* L858R mutation in cis. There is still no clear evidence to guide the therapeutic options for patients with both *EGFR* T790M and T854A mutations.

**Patient concerns:**

A 60-year-old Chinese woman with no smoking history presented with a maximum diameter of 32.9 mm mass located in the right lower lung lobe.

**Diagnosis:**

The patient was diagnosed with stage IVA lung adenocarcinoma with an exceptionally uncommon *EGFR* T854A mutation in exon 21 was detected concomitantly with *EGFR* T790M in blood by next-generation sequencing (NGS).

**Interventions:**

The patient was initially treated with first-line afatinib. After disease progression, osimertinib was administered.

**Outcomes:**

Our patient exhibited a partial response (PR) to osimertinib with progression-free survival of nearly 8 months.

**Conclusions:**

Our study indicates that patients with NSCLC who are positive for uncommon *EGFR* T854A and T790M mutations might benefit from treatment with osimertinib.

## 1. Introduction

Epidermal growth factor receptor (*EGFR*) T854A mutation in exon 21 is an uncommon *EGFR* mutation in patients with non-small cell lung cancer (NSCLC).^[1]^ It was reported that *EGFR* T854A is a secondary *EGFR* mutation after first- and second-generation EGFR tyrosine kinase inhibitors (TKIs).^[2,3]^ The resistance mechanism of *EGFR* T854A is similar to T790M, substitution produces an enhanced affinity for ATP, thus further reducing the ability of ATP-competitive reversible EGFR TKIs to bind to the tyrosine kinase domain of EGFR. The third-generation EGFR-TKI osimertinib (irreversible EGFR inhibitors) has been shown to be effective against common *EGFR* mutations and *EGFR* T790M mutation.^[4,5]^ In a previous study, all *EGFR* T854A mutations were co-occurred with *EGFR* L858R mutation in cis.^[6]^ However, there is still no clear evidence to guide the therapeutic options for patients with both *EGFR* T854A and T790M mutations.

In this study, we reported a case with advanced-stage NSCLC harboring a rare secondary *EGFR* T854A compound mutation, in which the third-generation EGFR-TKI osimertinib elicited durable responses.

## 2. Case presentation

**Figure 1.**
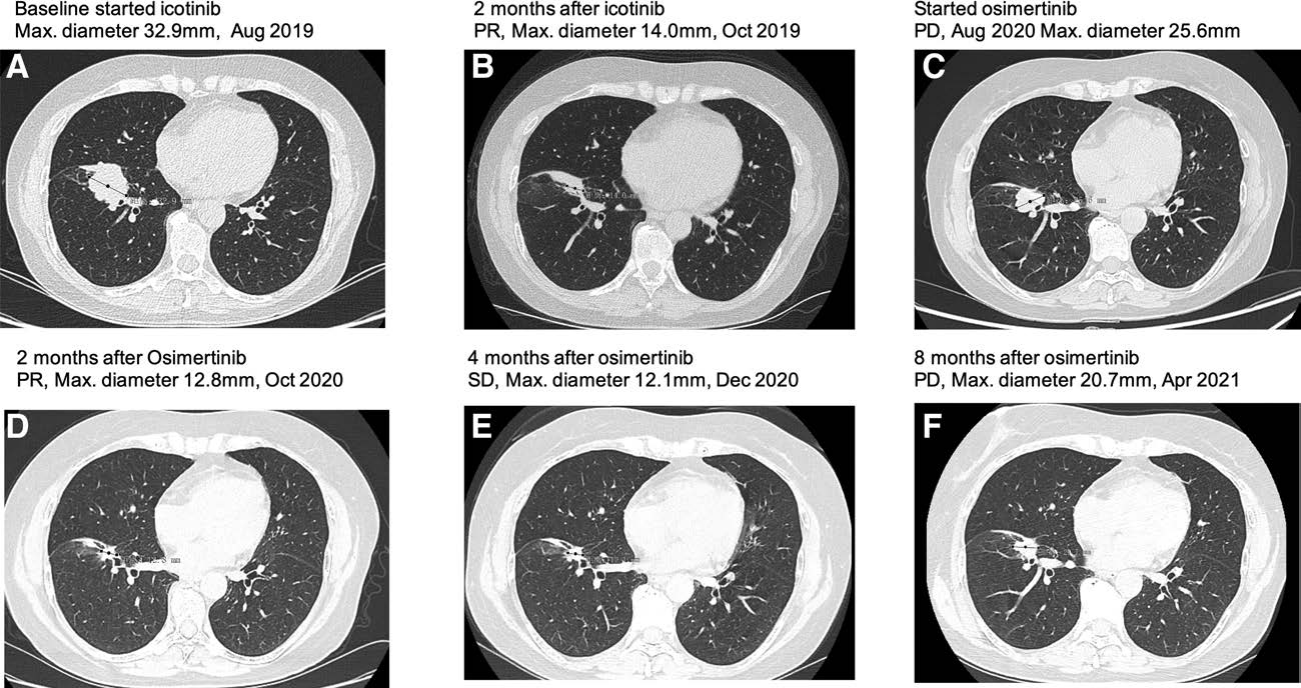
The CT images of the primary lung mass at baseline (A), at the evaluation of PR after 2 months of icotinib treatment (B), at the evaluation of PD after12 months of afatinib treatment (C), at the evaluation of PR after 2 months of osimertinib treatment (D), at the evaluation of SD after 4 months of osimertinib treatment (E), and at the evaluation of PD after 8 months of osimertinib treatment (F). CT = computed tomography, SD = stable disease, PR = partial response, PD = progressive disease.

A 60-year-old Chinese woman with no smoking history presented to our hospital. A chest contrast-enhanced computed tomography (CT) scan revealed a maximum diameter of 32.9 mm mass located in the right lower lung lobe (baseline, Fig. 1A). A bone scan showed left ilium metastasis. After a comprehensive evaluation, the patient was diagnosed with stage IVA lung adenocarcinoma with bone metastasis. Next-generation sequencing (NGS) performed on formalin-fixed paraffin-embedded (FFPE) samples of lung lesions indicated the presence *of EGFR* mutation (E746_T751delinsVP) with mutation frequencies of 20.06%. The patient was administered with icotinib as a first-line treatment at 125 mg orally tid in August, 2019. From a CT scan in October, 2019, the patient was assessed as partial response (PR) based on the Response Evaluation Criteria on Solid Tumors version (RECIST) version 1.1 (Fig. 1B). After 12 months of icotinib treatment, chest CT scans revealed a marked increase in tumor size that led to progressive disease (PD, maximum diameter 25.6 mm; Fig. 1C). The progression-free survival (PFS) of icotinib was 12 months.

A second targeted sequencing performed on blood by DNA-based NGS assay (Burning Rock OncoScreen Plus®, Burning Rock Biotech, Guangzhou, China), indicated the presence of *EGFR* mutations T790M, T854A and E746_T751delinsVP. The treatment was then switched to osimertinib at 80 mg once daily. After another 2 months of treatment, CT scans revealed remarkable tumor shrinkage of the left lung mass (Fig. 1D). The patient achieved PR. After 4 months of treatment, CT scans revealed slight tumor shrinkage of the left lung mass (Fig. 1E). The patient achieved stable disease (SD). After 8 months of osimertinib treatment, chest CT scans revealed a marked increase in tumor size that led to PD (Fig. 1F). The PFS of osimertinib was 8 months.

**Figure 2.**
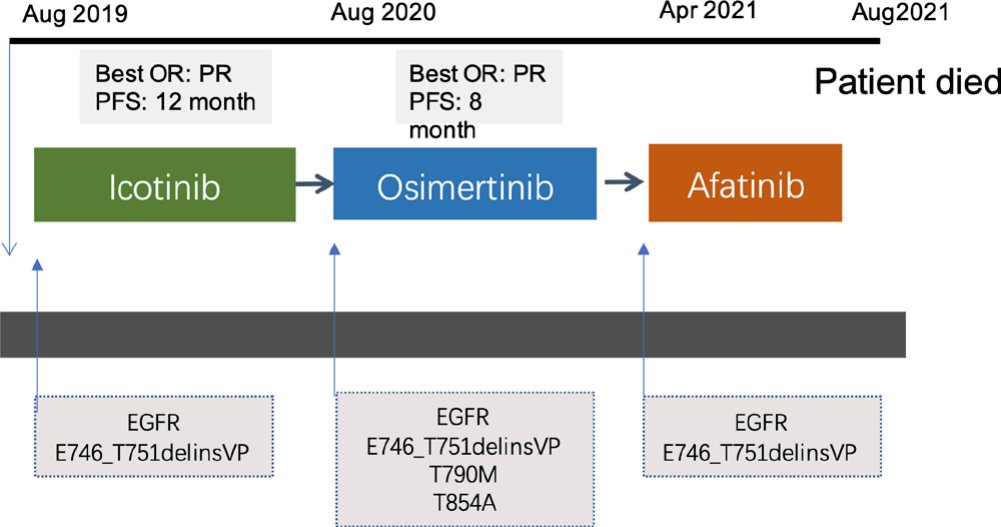
Timeline of the patient’s treatment history. OR = best overall response, PFS = progression-free survival, PR = partial response.

The third targeted sequencing performed on the blood by DNA-based NGS assay (Burning Rock OncoScreen Plus®, Burning Rock Biotech, Guangzhou, China), indicated the presence of *EGFR* mutation E746_T751delinsVP. In April, 2021, a decision was made to challenge afatinib. At 1-week follow-up, the patient experienced serious adverse effects. The patient’s clinical symptoms were getting worse. On August 27th, 2021, the patient died. A timeline of the patient’s treatment history was presented in Figure 2.

All procedures performed in studies involving human participants were in accordance with the ethical standards of the institutional and/or national research committee(s) and with the Helsinki Declaration (as revised in 2013). Written informed consent was obtained from the patient’s legal guardian/next of kin for the publication of this manuscript and any accompanying images.

## 3. Discussion

*EGFR* T854A mutation is often acquired after resistance to first-generation EGFR TKIs. And it is often detected in patients with *EGFR* L858R mutation. No effective targeted therapeutic option has been proposed for patients with *EGFR* T854A mutation co-current with T790M mutation. This case reported the durable clinical efficacy of osimertinib in a patient with metastatic NSCLC carrying secondary mutations *EGFR* T854A co-current with T790M mutations.

The most common secondary mutation found in lung adenocarcinomas after first or second-generation TKIs was the *EGFR* T790M mutation in exon 20 (accounting for 50%–60%).^[7,8]^ *EGFR* T854A mutation is rare, accounting for 0.06% of Chinese NSCLC patients. Structurally, T854A mutation is at the “bottom” of the ATP-binding pocket (drug contact site),^[2]^ suggesting the amino acid substitutions alter drug contact residues in the *EGFR* ATP-binding pocket and lead to resistance to first-generation TKIs (gefitinib). Previous in vitro studies indicated Ba/F3 cell lines with uncommon secondary mutations (*EGFR* L858R/T854A) were sensitive to the irreversible third-generation EGFR-TKIs, indicating that third-generation EGFR-TKIs treatment might also be effective for the treatment of patients with uncommon secondary *EGFR* T854A mutations.^[3]^

In a retrospective cohort study, Zhang et al reported a Chinese 50-year-old man diagnosed with lung adenocarcinoma stage IV. The patient was initially treated with icotinib. When the disease progressed, he was then switched to osimertinib treatment when NGS indicated the presence of *EGFR* T854A, T790M and E746_A750del (exon 19) mutations. This patient achieved the PR with a PFS time of 4 months. This case agreed with our study, that the inhibitory activity of osimertinib is able to overcome resistance to first-generation EGFR TKIs, as indicated by notable tumor shrinkage in these two patients.

## 4. Conclusion

Our case suggested that osimertinib is a promising treatment strategy for managing patients with metastatic NSCLC carrying secondary mutations *EGFR* T854A co-current with T790M mutations.

## Acknowledgments

The authors would like to thank Dr Xi Li from Burning Rock Biotech for their suggestions in data analysis and manuscript writing.

## Author contributions

**Conceptualization:** Hongbin Sun, Nan Zhao.

**Data curation:** Nan Zhao, Zhiqi Li.

**Formal analysis:** Hongbin Sun.

**Investigation:** Hongbin Sun, Nan Zhao, Hua Xin, Changjuan Qin, Zhiqi Li.

**Methodology:** Hua Xin, Changjuan Qin, Zhiqi Li.

**Project administration:** Hua Xin.

**Resources:** Hongbin Sun.

**Supervision:** Changjuan Qin, Hongbin Sun.

**Writing—original draft:** Changjuan Qin, Hongbin Sun, Nan Zhao, Hua Xin, Zhiqi Li.

**Writing—review & editing:** Hongbin Sun, Nan Zhao, Hua Xin.
